# Prenatal diagnosis and clinical management of cardiac rhabdomyoma: a single-center study

**DOI:** 10.3389/fcvm.2024.1340271

**Published:** 2024-02-16

**Authors:** Longzhuang Peng, Youchun Cai, Jianhang Wu, Wen Ling, Qiumei Wu, Shan Guo, Biying Huang, Caihong Jiang, Zongjie Weng

**Affiliations:** Department of Medical Ultrasonics, Fujian Maternity and Child Health Hospital, College of Clinical Medicine for Obstetrics & Gynecology and Pediatrics, Fujian Medical University, Fuzhou, China

**Keywords:** fetal cardiac rhabdomyoma, tuberous sclerosis complex, ultrasound, genetic testing, prognosis

## Abstract

**Objective:**

The study aims to assess the ultrasonic features of fetal cardiac rhabdomyoma (CR), track the perinatal outcome and postnatal disease progression, investigate the clinical utility of ultrasound, MRI and tuberous sclerosis complex (TSC) gene analysis in CR evaluation, and offer evidence for determing of fetal CR prognosis.

**Methods:**

We conducted a retrospective analysis of prenatal ultrasound-diagnosed fetal CR cases in our hospital from June 2011 to June 2022, tracked the perinatal outcomes, regularly followed live infants to analyze cardiac lesion changes and disease progression, and compared the sensitivities of ultrasound, MRI and their combination in the detecting of intracranial sclerosing nodules.

**Results:**

Our study included 54 fetuses with CR: 32 pregnancies were terminated, 22 were delivered, 35 were diagnosed with TSC, 13 had simple CR without TSC, and in 6 cases, remained unclear whether TSC accompanied the CR due to insufficient evidence. 45 fetuses (83.3%) had multiple lesions, while 9 fetuses (16.7%) presented with a single lesion. Twelve cases had intracardiac complications, all associated with multiple lesions, and these cases exhibited larger maximum tumor diameters than the non-complicated group. Multiple intracardiac lesions were more prevalent in the TSC group than in the simple CR group. However, there was no significant difference in maximum tumor diameter between the two groups. Among 30 fetuses who underwent fetal brain MRI, 23 were eventually diagnosed with TSC, with 11 fetuses showing intracranial sclerosis nodules by ultrasound and 15 by MRI, and the diagnostic consistency was moderate (*k* = 0.60). Twenty-two fetuses were born and followed up for 6–36 months. CR lesions diminished or disappeared in 18 infants (81.8%), while they remained unchanged in 4 infants (18.2%). Ten out of 12 (83.3%) surviving children diagnosed with TSC developed epilepsy, and 7 (58.3%) had neurodevelopmental dysfunction.

**Conclusions:**

The majority of CR cases involve multiple lesions, which are a primary risk factor for TSC. Through prenatal ultrasound examination is crucial for assessing fetal CR prognosis. Although ultrasound combined with MRI can detect intracranial sclerosis nodules in TSC fetuses, its sensitivity is limited. TSC gene sequencing is an essential diagnostic method. Simple CR cases without TSC generally have a favorable prognosis.

## Introduction

Cardiac rhabdomyoma (CR) is one of the most common primary cardiac tumors, accounting for approximately 60% of the various primary cardiac tumors in children (1, 2). Its incidence in live-born infants ranges from 0.02% to 0.17%, with a prevalence of 0.12% in prenatal fetuses (3, 4). CR is characterized as a benign hamartomatous tumor. In cases where CR lesions do not lead to severe complications, the pregnancy outcome is generally favorable (5). However, complications associated with CR can include arrhythmias, valvular regurgitation, outflow tract obstruction, heart failure, pericardial effusion, fetal edema, and, rarely, stillbirth (3, 6).

CR serves as a significant clinical marker and initial symptom of tuberous sclerosis complex (TSC) (7–9), with a high risk of neurodevelopmental impairment. According to previous studies, the incidence of TSC in fetuses with CR ranges from 50% to 90% (10, 11). Moreover, approximately 90% of infants diagnosed with TSC typically experience infantile seizures, intellectual impairment, or autism (12). TSC is a multilineage-based neurocutaneous syndrome that is an autosomal dominant disorder caused by mutations in the TSC1 and TSC2 genes. While around two-thirds of TSC cases are sporadic, the remaining third have familial origins (8, 13). Cortical and subependymal nodules (SENs) are hallmark brain lesions in TSC and act as sensitive indicators for TSC diagnosis. Studies have shown that nearly all individuals with TSC display pathological changes in the nervous system, with 80%–90% having cortical nodules and/or SENs (14). Notably, there appears to be no significant distinction in pathogenic mutations between TSC1 and TSC2. Furthermore, it has been reported that neuroimaging findings are more sensitive indicators of TSC and correlate with a less favorable prognosis (15). Therefore, the prenatal detection of intracranial lesions is a pivotal component for diagnosing TSC in fetuses exhibiting CR.

Due to the correlation between CR and TSC, diagnosing and assessing CR in pregnant women is crucial. Clinical symptoms of TSC vary widely, and genetic testing for TSC can be costly and not widely recommended (16, 17). Heart and brain abnormalities, especially in the prenatal and early postpartum periods, are often the only signs of TSC (17). Fetal echocardiography is the primary method for detecting cardiac tumors (18). While it's effective for CR, ultrasound's sensitivity in identifying brain lesions in TSC fetuses is unclear. Fetal brain MRI is preferred for diagnosing neurological issues, which complements and confirms the diagnosis of TSC with genetic testing (15). The effectiveness of ultrasound compared to MRI for intracranial nodules remains underexplored. In addition, the connection between TSC and multiple CRs is recognized, but the association with a single CR remains is uncertain (19, 20), making prognostic assessment challenging.

Detecting CR in fetus is essential for assessing short-term perinatal outcomes and long-term prognostic considerations in the context of TSC. Previous studies often focused on prenatal diagnosis or postnatal treatment and prognosis, and rare disease studies require substantial sample size. we analyzed the prenatal and postnatal data of 54 CR fetuses which was diagnosed by prenatal ultrasound. Our goal was to evaluate perinatal risks and prenatal diagnosis of fetuses presenting with CR as the initial symptom, using a multidisciplinary approach. We aimed to determine the clinical value of combining ultrasound and MRI with TSC gene detection for assessing CR fetus prognosis, providing guidance for perinatal management and prognostic evaluation.

## Materials and methods

### Clinical characterization

We retrospectively analyzed data from 75 fetuses with CR out of 124,589 pregnant women who underwent prenatal ultrasound examinations at Fujian Maternal and Child Health Hospital between June 2011 and June 2022. Our analysis included ultrasonography, brain MRI, genetic testing, pathologic anatomy and medical records of surviving infants. Fetuses with CR underwent systematic ultrasonography were verified through our diagnostic methods. Exclusion criteria comprised cases with cardiac tumor which could not be determined as CR, incomplete clinical data, or a lack of follow-up. After excluding 21 cases due to insufficient diagnostic evidence or incomplete data, the study included 54 cases. The study protocol was reviewed and approved by the Ethics Committee of the Fujian Maternal and Child Health Hospital, with informed consent from all pregnant women.

### Fetal ultrasonography

GE Voluson E8 and E10 high-resolution color Doppler ultrasound diagnostic instruments were employed, with a frequency of the transabdominal ultrasound probe of 4∼8 MHz. The conditions of middle and late pregnancy and fetal heart examination were selected, and transvaginal examination was performed to observe the fetal intracranial structure, with a frequency of the cavity probe of 6∼10 MHz. Neonatal echocardiography for live births employed a Phillips EPIQ 7C with 3∼8 MHz phased array probes. All assessments followed International Society of Ultrasound in Obstetrics and Gynecology (ISUOG) guidelines for prenatal ultrasonography (21, 22). Our examinations included the number, location, size, shape, calcification, capsule, liquefaction and pedicle formation of the tumor for diagnosis and differential diagnosis. We also checked for other cardiac malformations and complications, and conducted craniocerebral ultrasound examinations.

### Fetal brain MRI

We utilized a GE 1.5 Tesla MR Scanner with gradient field strength and phased line loop channels. The fetal brain scans included SSFSE and FIESTA sequences in standard cross-sections, coronal, and sagittal planes, along with DWI and T 1 fat-pressing sequences in cross-sections. The scanning time for all sequences did not exceed 15 min.

### Genetic analysis

For fetuses with CR detected via ultrasound, it is advisable to undergo TSC gene testing. Medical professionals with qualifications in prenatal diagnosis evaluated the need for prenatal testing. Informed consent from pregnant women and their families was obtained for procedures such as amniocentesis, umbilical vein puncture, and the amniotic fluid or umbilical cord blood extraction for karyotype analysis, single-nucleotide polymorphism comparative genomic hybridization or exon sequencing. The diagnosis of TSC was based on the diagnostic criteria of the International Tuberous Sclerosis Conference (23).

### Follow-up

In cases of pregnancy termination, anatomical and pathological examination were conducted to verify the diagnosis after obtaining informed consent and signature from patients and their family members. For newborns with cardiac tumors in our hospital, CR was confirmed through repeated clinical follow-up after birth. Children diagnosed with TSC underwent skin and eye examinations, abdominal ultrasonography, assessment of seizures, and brain MRI. Additionally, evaluations were performed for mental and psychomotor development, as well as behavioral and language skills. All children in the study received regular followed up by a pediatrician and an ultrasound physician. Those who declined to participate or could not be reached more than three occasions were considered lost to follow-up.

### Statistical analysis

IBM SPSS Statistics 26 and MedCalc 15.2.2 software were used for statistical analysis of the data. The Shapiro‒Wilk test was used to test the normality of the measurement data, the *t* test was used for comparisons between groups, and the Mann‒Whitney *U* test was used for comparisons between groups. The number of cases (%) was used to represent the count data, and the comparison between groups was performed by the *χ*^2^ test or Fisher's exact test. *P* < 0.05 was considered to indicate statistical significance. The kappa test was used for consistency checking. By plotting receiver operating characteristic (ROC) curves, the area under the curve (AUC), sensitivity, specificity, positive predictive value (PPV) and negative predictive value (NPV) were calculated to evaluate the diagnostic efficacy of each diagnostic method.

## Results

**Figure 1:**
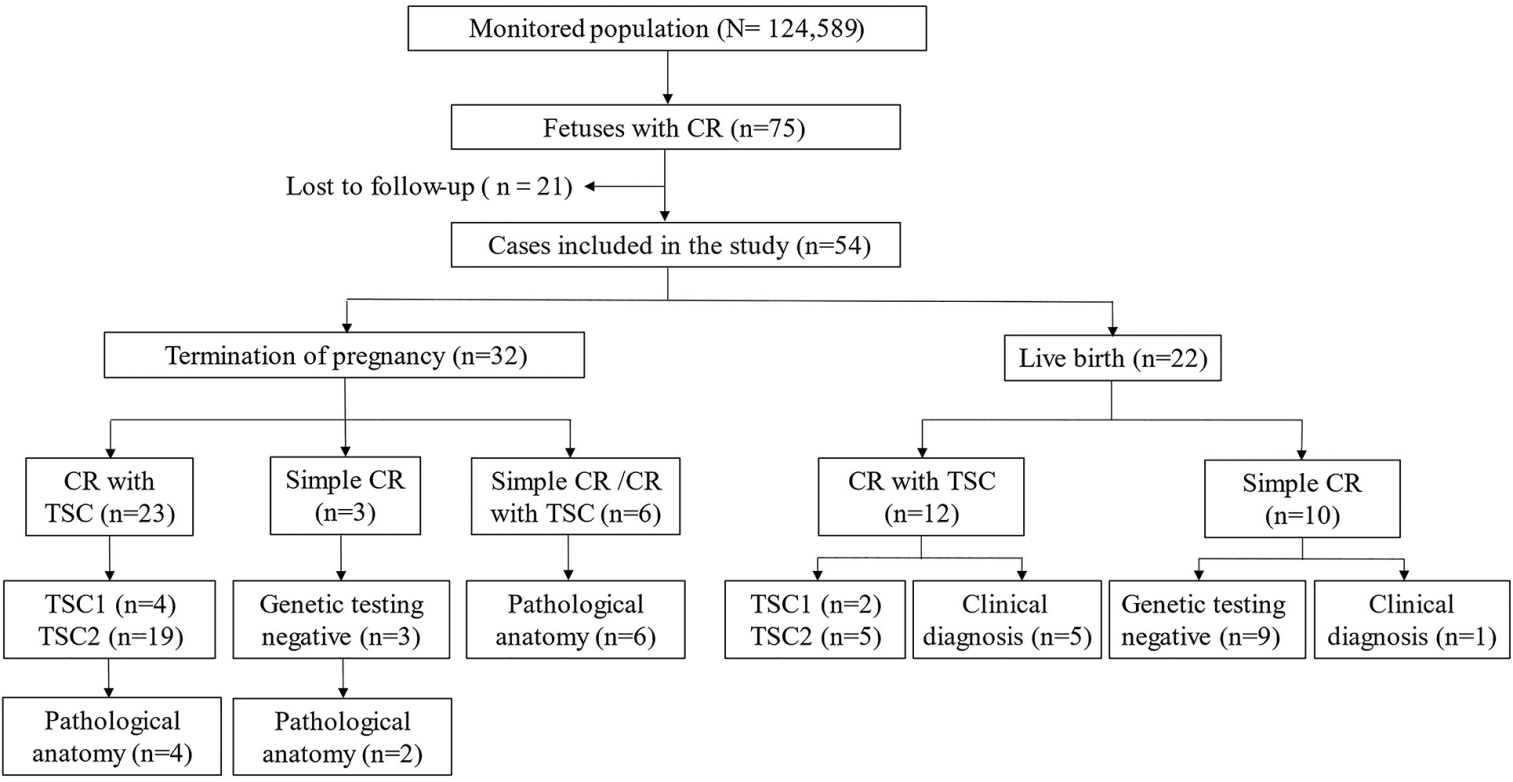
Flowchart of this retrospective study.

The study involved 54 pregnant women, with an average age of 28.04 ± 4.01 years (17–39 years) and an average gestational age of 28.91 ± 4.14 weeks (23–40 weeks). Thirty fetuses underwent both ultrasound and brain MRI examinations. Among the 22 fetuses born, 12 were diagnosed with TSC posnatally,8 with TSC1/2 gene mutations, and 4 based on clinical and imaging assessments by pediatric experts. Ten live-born children had simple CR without TSC or TSC gene mutations (TSC gene mutations or TSC-related clinical manifestations during follow-up). Among 32 induced pregnancies, 26 were tested for the TSC gene mutations, with 23 positive and 3 negative results. Autopsy and pathological examination diagnosed 12 cases with CR, including 4 with TSC1/2 gene mutations. In summary, 35 cases were CR with TSC, 13 were CR without TSC (named “simple CR” in this paper), and 6 had confirmed as CR, with TSC status remaining unclear due to insufficient evidence (Figure 1).

### Sonographic characterization of fetal CR

**Figure 2:**
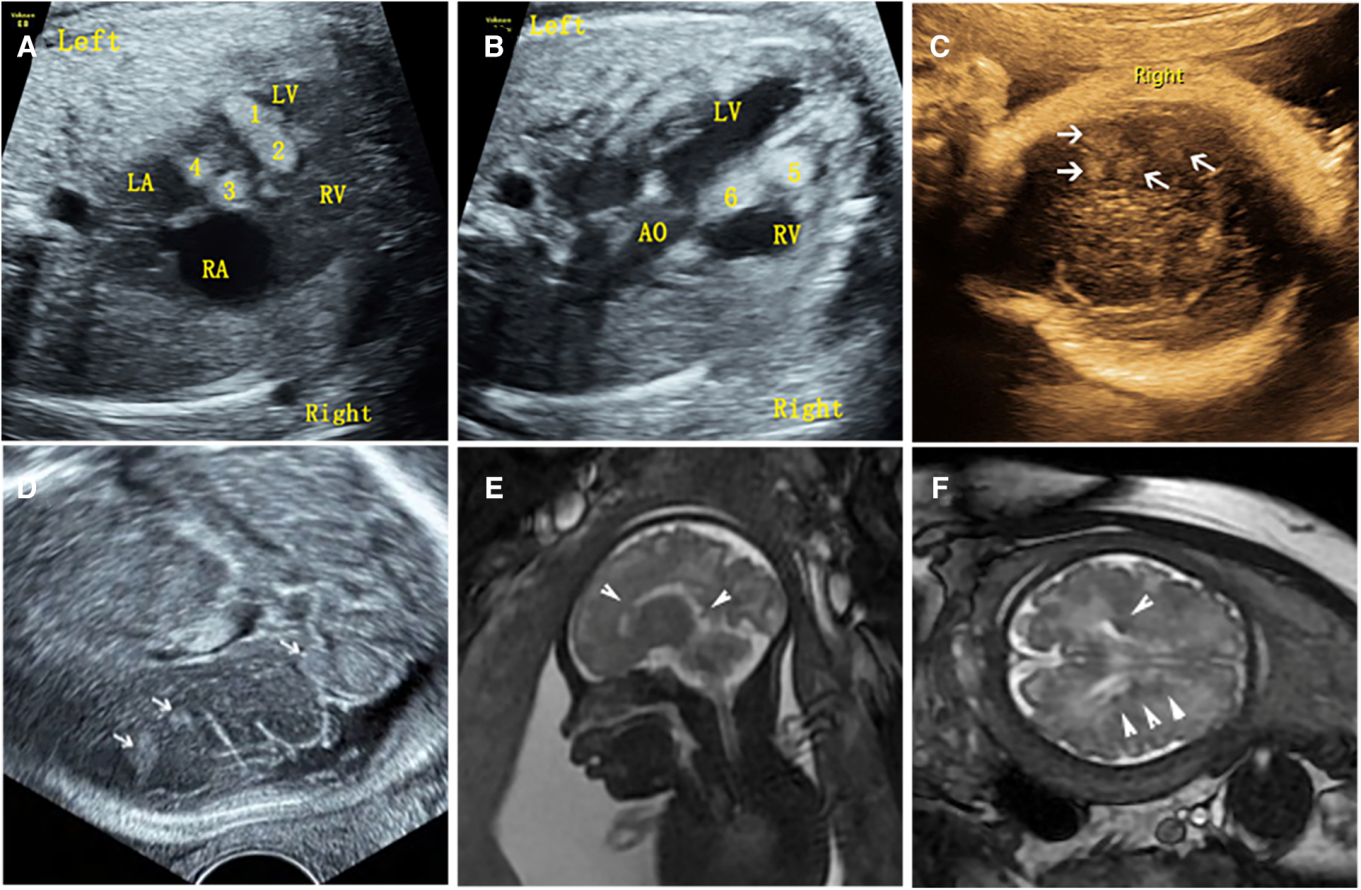
Ultrasound and MRI findings of multiple cardiac rhabdomyomas complicated with multiple cranial sclerotic nodules in the fetus (case NO. 51) at 31 weeks of gestation. (**A**) Multiple slightly hyperechoic nodules in the left ventricle of the fetal heart. (**B**) Multiple slightly hyperechoic nodules in the right ventricle of the fetal heart. (**C**) Multiple hyperechoic intracranial nodules of the fetus detected by transvaginal sonography. (**D**) Multiple hyperechoic intracranial nodules of the fetus detected by transabdominal sonography. (**E**) MRI sagittal T2-weighted image with multiple subependymal nodules. (**F**) MRI axial T2-weighted image with multiple subependymal nodules.

**Figure 3:**
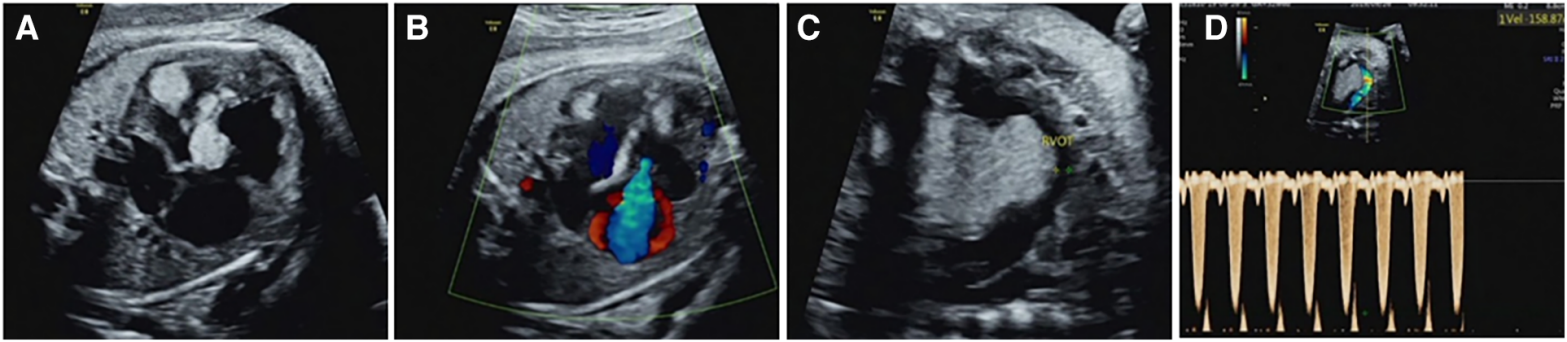
Intracardiac complications associated with cardiac rhabdomyoma in the fetus (case NO. 54) at 33 weeks of gestation. (**A**) Heart failure with cardiothoracic ratio enlargement. (**B**) Severe tricuspid regurgitation. (**C**) Rhabdomyoma located in the upper interventricular septum protrudes into the right ventricular outflow tract, resulting in a reduced internal diameter of the right ventricular outflow tract. (**D**) Increased velocity of the right ventricular outflow tract.

54 cases of fetal CR typically displayed round, homogeneous, high/slightly hyperechoic, intramural or intraluminal masses (Figure 2). Most cases (83.3%) had multiple lesions, with an average maximum tumor diameter of 12.26 ± 6.23 mm. Lesions were primarily found in the left ventricle (81.5%) and right ventricle (77.8%), while some appeared in the ventricular septum (42.6%), right atrium (14.8%), and left atrium (1.9%). Around 22.2% of fetuses had intracardiac complications, mainly outflow tract obstruction, arrhythmia, heart failure, pericardial effusion, or severe tricuspid regurgitation (Figure 3). The fetuses with intracardiac complications all had multiple lesions. Five fetuses had outflow tract obstruction because the tumors were located near the upper part of the ventricular septum. The mean maximum CR diameter was significantly larger in the group with intracardiac complications (17.92 ± 8.43 mm) compared to the group without complications (10.64 ± 4.37 mm) with a *p*-value of 0.000. This highlights the importance of lesion quantity, location, and size in causing fetal cardiac complications.

### Comparison of fetal brain ultrasound and MRI

**Figure 4:**
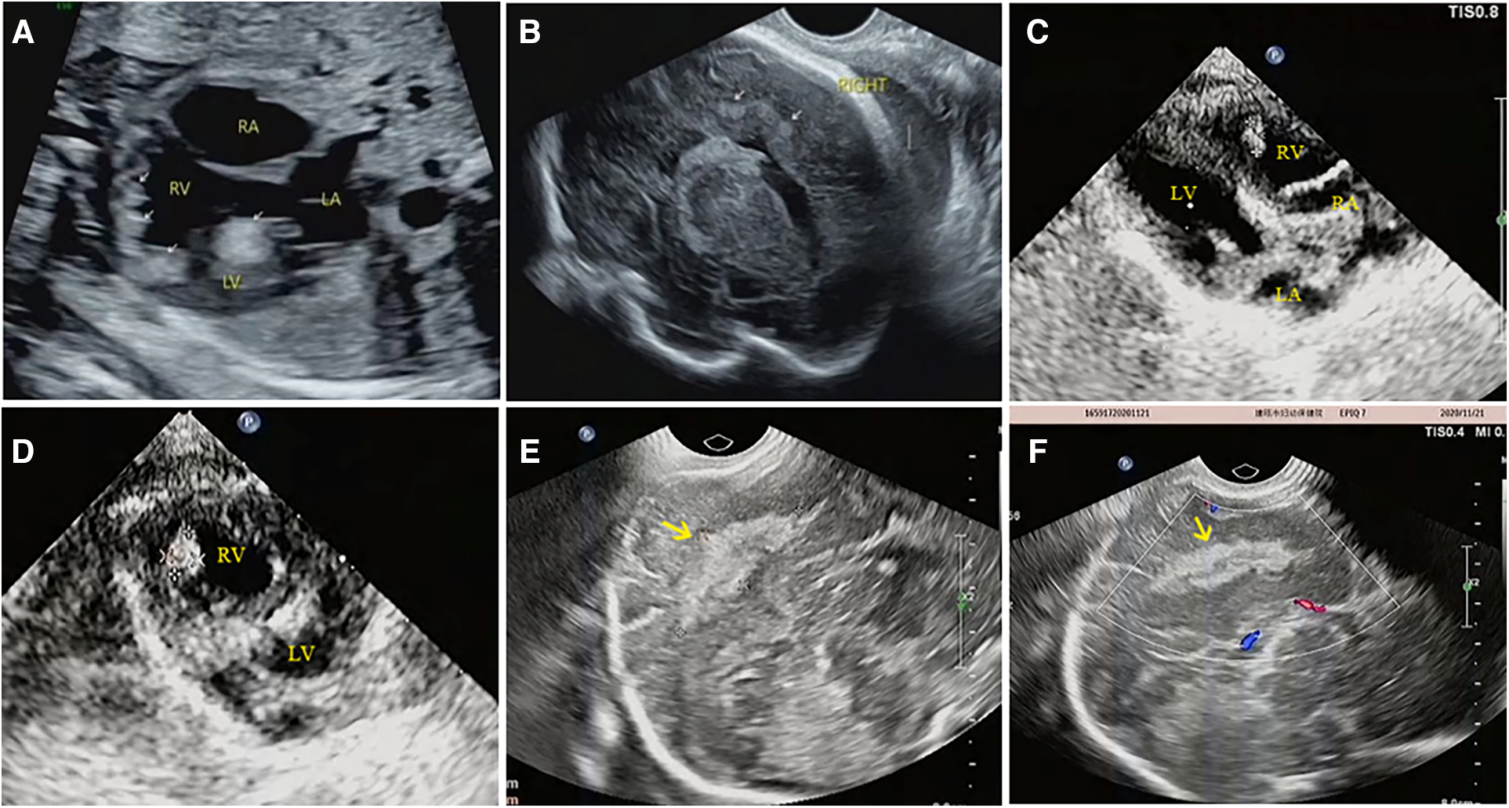
Prenatal and postnatal ultrasonography of cardiac rhabdomyoma complicated with cranial sclerotic nodules in case NO. 13. (**A**) Multiple slightly hyperechoic nodules in the ventricle of the fetal heart at 28 weeks of gestation. (**B**) Multiple hyperechoic intracranial nodules of the fetal heart at 28 weeks of gestation; however, no intracranial lesions were detected by MRI at the same time. (**C**,**D**) Multiple slightly hyperechoic nodules in the ventricle of the baby at 1 month after birth. (**E**,**F**) Hyperechoic intracranial nodule of this baby at the same time.

**Figure 5:**
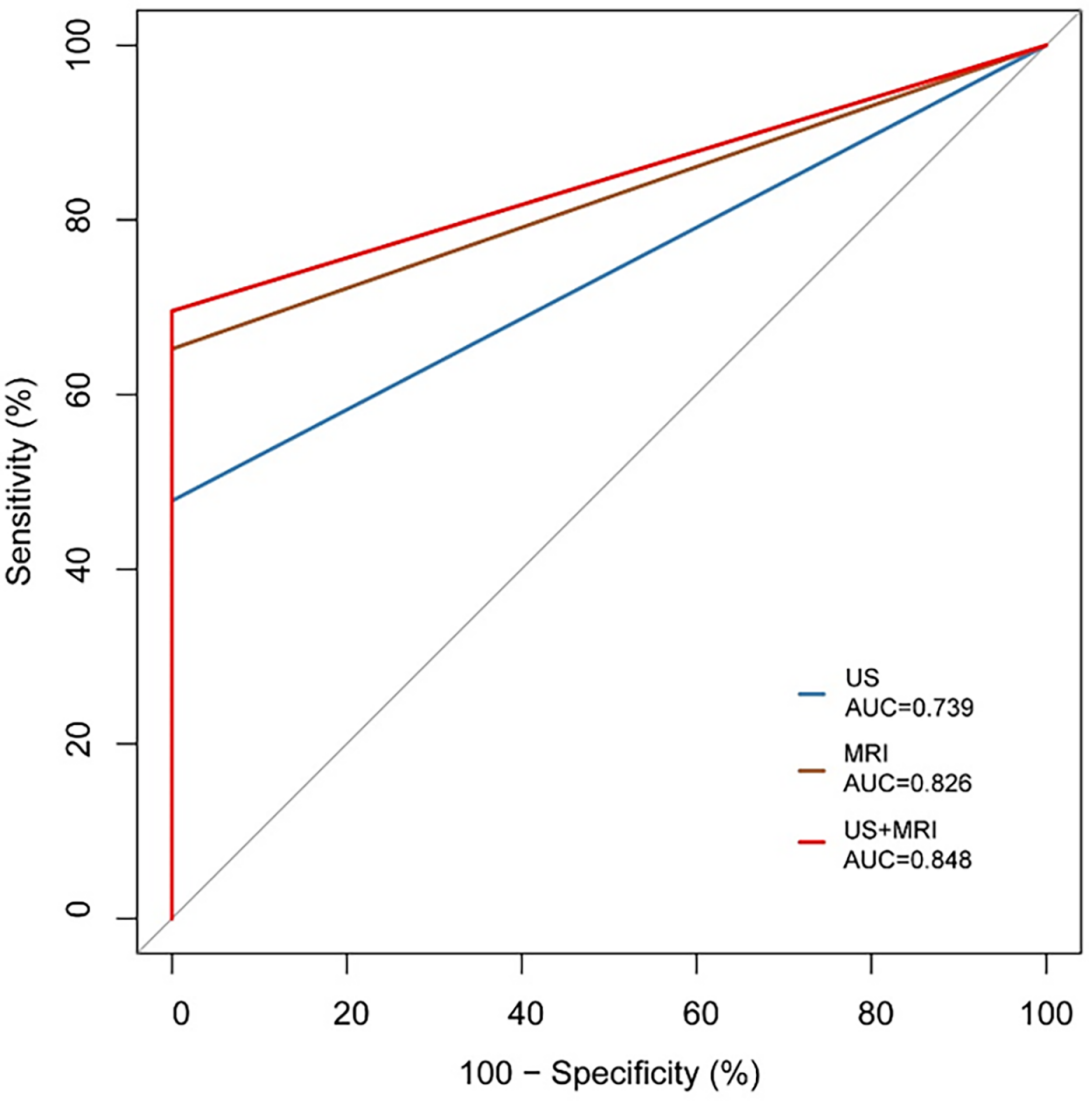
Comparison of the ROC of ultrasound and MRI in diagnosing TSC.

Of the 54 fetuses with CR diagnosed by ultrasound, 30 underwent fetal MRI. On ultrasound, 11 cases displayed intracranial nodules, often appearing as hyperechoic or slightly hyperechoic nodules, typically occurring in multiples (Figure 2). The majority (90.9%) were in the brain parenchyma, and some (27.3%) in the subependymal area. Prenatal brain MRI confirmed 15 cases of intracranial sclerosing nodules, which displayed a high signal on T1 and a low signal on T2 (Figure 2). A substantial portion (40%) was located in the brain parenchyma, while the majority (93.3%) were in the subependymal area. One case showed multiple intracranial lesions on ultrasound but no MRI abnormalities (Figure 4); postnatal MRI later confirmed intracranial nodules. Ultrasound combined with MRI identified 16 fetuses with intracranial sclerosing nodules, with 14 having TSC1/2 gene mutations, and 2 diagnosed with Tuberous Sclerosis Complex (TSC) postnatally. Among 14 fetuses without prenatal intracranial nodules, 7 had TSC1/2 gene mutations, 6 tested negative for the TSC gene, and 1 did not undergo TSC genetic testing, being diagnosed with simple CR after a 5-year follow-up. Moderate agreement was seen between ultrasound and MRI in diagnosing TSC (K = 0.60). Sensitivity for ultrasound, MRI, and the combination of both in predicting CR with TSC was 47.83, 65.22, and 69.57, respectively (Table 1, Figure 5). The corresponding AUC values were 0.739, 0.826, and 0.848, with MRI demonstrating higher sensitivity in detecting intracranial sclerosing nodules in fetuses with CR. No significant difference in AUC values was observed among the three groups (*P* > 0.05).

**Table 1:** Diagnostic performance of ultrasound and MRI in diagnosing TSC.

Diagnostic method	AUC	Sensitivity (%)	Specificity (%)	Positive predictive value (%)	Negative predictive value (%)
US	0.739 (0.547, 0.881)	47.83 (26.8, 69.4)	100 (85.2, 100.0)	76.7 (57.7, 90.1)	36.8 (16.3, 61.6)
MRI	0.826 (0.644, 0.939)	65.22 (42.7, 83.6)	100 (85.2, 100.0)	76.7 (57.7, 90.1)	46.7 (21.3, 73.4)
US&MRI	0.848 (0.670, 0.952)	69.57 (47.1, 86.8)	100 (85.2, 100.0)	76.7 (57.7, 90.1)	50 (23.0, 77.0)

### Pathological anatomy of CR

**Figure 6:**
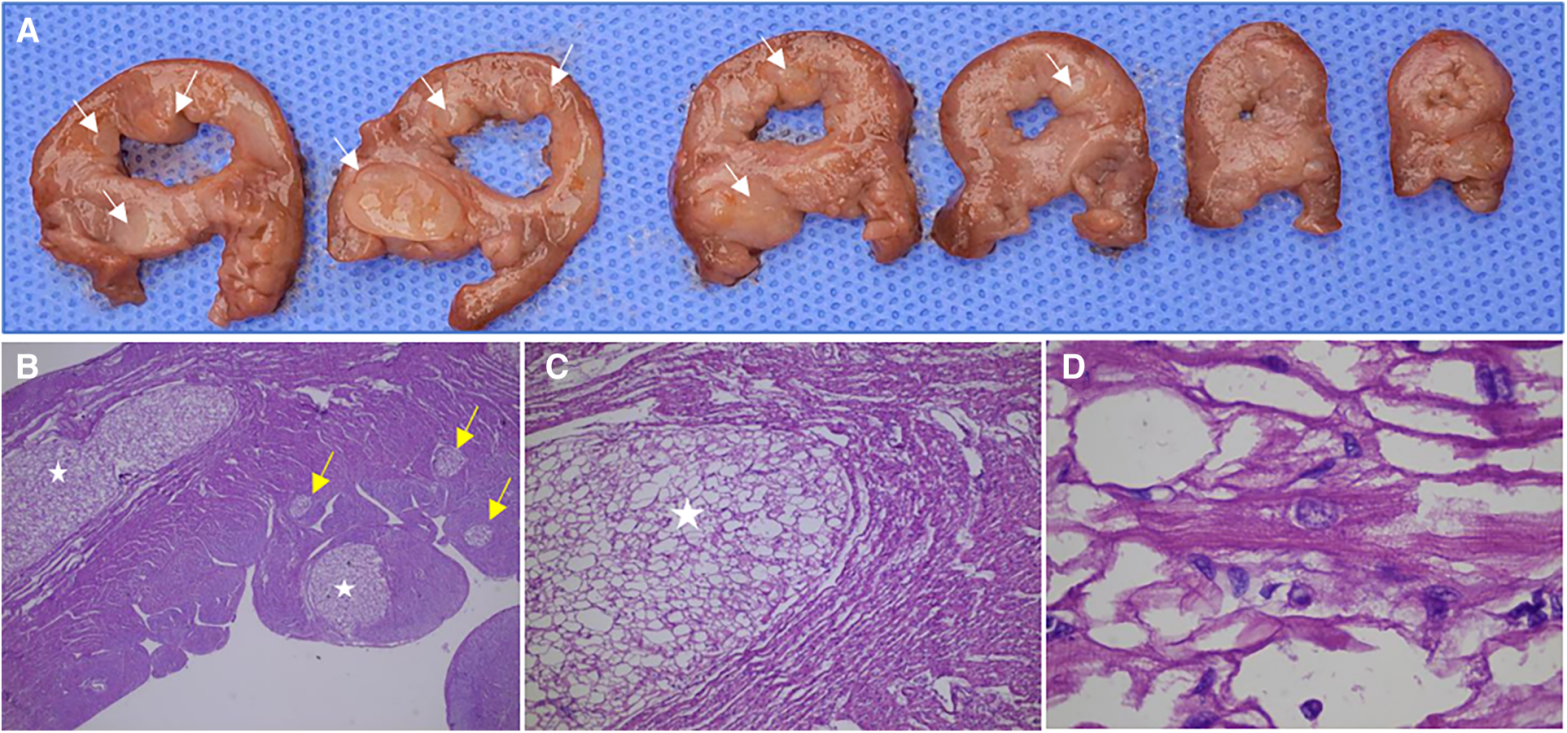
Anatomical and pathological specimens of a fetus (case NO. 51) terminated at 31 weeks of gestation. (**A**) Multiple rhabdomyomas in the left ventricle and interventricular septum were observed in continuous transverse sections. (**B**) The sections were stained by HE and observed by ×25 magnification, showing multiple intramyocardial rhabdomyomas, some of which were only 1–3 mm in diameter (yellow arrow). (**C**) The sections were stained by HE and observed by ×50 magnification, showing a rhabdomyoma in the myocardium. (**D**) The sections were stained by HE and observed by ×250 magnification, showing the cell morphology and arrangement in rhabdomyoma. The cardiac tumors were composed of irregular and swollen cardiomyocytes with vacuolated cytoplasm.

Of the 54 fetuses, 32 pregnancies were terminated, none of them with other malformations, and 12 were confirmed to have Cardiac Rhabdomyomas (CR) through anatomical and pathological examination. Pathological analysis showed that these cardiac tumors were composed of irregular, swollen cardiomyocytes with vacuolated cytoplasm (Figure 6). In three cases, pathology identified more rhabdomyomas than were initially diagnosed by prenatal ultrasound, with these lesions located within the ventricular wall, often measuring less than 5 mm in diameter. Many of the lesions were only 1–2 mm, making them challenging to detect via ultrasound.

### Comparison of heart lesions between the TSC group and simple CR group

Multiple lesions were notably more common in the TSC group (32/35, 91.4%) than in the simple CR group (7/13, 53.8%) with a *p*-value of 0.007, suggesting a higher likelihood of multiple Cardiac Rhabdomyomas (CR) in conjunction with Tuberous Sclerosis Complex (TSC). However, the mean maximum tumor diameter did not significantly differ between the two groups, measuring 11.94 ± 5.64 mm in the TSC group and 11.15 ± 6.00 mm in the simple CR group (*p* = 0.672). Of the 22 live births, 12 were diagnosed with TSC, while 10 were diagnosed with simple CR. These cases were followed up with echocardiography for 3 to 36 months. Among the 22 live births, 27.3% (6/22) experienced tumor disappearance, 54.5% (12/22) saw regression, and 18.2% (4/22) showed no change. In the 12 TSC cases, the rates were 25.0% (3/12), 58.3% (7/12), and 16.7% (2/12), respectively, and in the 10 simple CR cases, the rates were 30.0% (3/10), 50.0% (5/10), and 20.0% (2/10), respectively. No significant difference was observed between the TSC and simple CR groups (*P* = 1.000), shown in Table 2.

**Table 2:** Comparison of CR by ultrasound between the CR with TSC and simple CR groups.

	CR with TSC	Simple CR	
Number of tumors			
Single	3/35 (8.6%)	6/13 (46.2%)	*P* = 0.007
Multiple	32/35 (91.4%)	7/13 (53.8%)	
Max diameter	11.94 ± 5.64 mm	11.15 ± 6.00 mm	*P* = 0.672
Postnatal change in size			
Progression	0/12 (0%)	0/10 (0%)	*P* = 1.000
No change	2/12 (16.7%)	2/10 (20.0%)	
Decrease	7/12 (58.3%)	5/10 (50.0%)	
Disappear	3/12 (25.0%)	3/10 (30.0%)	

### Survival Status and prognosis of live birth

Out of the 12 surviving patients diagnosed with Tuberous Sclerosis Complex (TSC), 10 (83.3%) experienced epilepsy, 4 (33.3%) had severe neurodevelopmental dysfunction, 3 (25.0%) had mild neurodevelopmental dysfunction, 9 (75.0%) had skin hypomelanotic macules, 3 (25.0%) had eye retinal hamartomas, 1 (8.3%) had eye retinal pigment degeneration, 2 (16.7%) had multiple renal cysts, and 1 (8.3%) had kidney angiolipomas. Clinical manifestations in live-born TSC patients are detailed in Tables 3 and 4. Ten children with simple Cardiac Rhabdomyomas (CR) were monitored for 6 months to 6 years, displaying good growth and development without TSC-related clinical symptoms. Among live births with prenatal intracardiac complications, one patient exhibited antepartum arrhythmias but had an average heart rate during the third trimester and after birth. Two patients had left ventricular outflow tract obstruction, with the number of lesions decreasing and the outflow tract obstruction gradually improving after birth.

**Table 3:** The clinical manifestations of live-born fetuses with TSC (*N* = 12).

Epilepsy	*n* (%)
Present	10/12 (83.3%)
Absent	2/12 (16.7%)
Neurodevelopmental status	
Severe dysfunction	4/12 (33.3%)
Mild dysfunction	3/12 (25.0%)
Normal	5/12 (41.7%)
Other organs disability	
Skin hypomelanotic macules	9/12 (75.0%)
Eyes retinal hamartomas	3/12 (25.0%)
Eyes retinal pigment degeneration	1/12 (8.3%)
Kidney angiolipomas	1/12 (8.3%)
Kidney multiple renal cysts	2/12 (16.7%)

**Table 4:** The outcomes of live-born children with cardiac rhabdomyoma.

Case	TSC	Diagnostic basis	Epilepsy	Other organs disability	Neurodevelopmental status	Family history	CR change after birth
1	No	Genetic	Absent	Absent	Normal	No	6M decrease
2	No	Genetic	Absent	Absent	Normal	No	10M disappear
3	Yes	Genetic	Present	Skin hypomelanotic macules; Eyes retinal pigment degeneration; Kidney multiple renal cysts	Normal	No	12M disappear
4	No	Genetic	Absent	Absent	Normal	No	36M no change
5	Yes	Genetic	Present	Absent	Severe dysfunction	No	36M disappear
6	Yes	Clinical	Present	Skin hypomelanotic macules	Mild dysfunction	No	3M disappear
7	Yes	Clinical	Present	Skin hypomelanotic macules	Severe dysfunction	Yes	12M decrease
8	No	Genetic	Absent	Absent	Normal	No	12M decrease
9	Yes	Clinical	Present	Absent	Severe dysfunction	No	18M decrease
10	Yes	Genetic	Present	Skin hypomelanotic macules	Severe dysfunction	Yes	12M decrease
11	No	Genetic	Absent	Absent	Normal	No	24M decrease
12	No	Genetic	Absent	Absent	Normal	No	12M disappear
13	Yes	Clinical	Present	Skin hypomelanotic macules	Normal	No	24M no change
14	No	Clinical	Absent	Absent	Normal	No	6M decrease
15	No	Genetic	Absent	Absent	Normal	No	3M disappear
16	Yes	Clinical	Present	Skin hypomelanotic macules; Eyes retinal hamartomas; Kidney multiple renal cysts	Normal	No	12M decrease
17	No	Genetic	Absent	Absent	Normal	No	24M decrease
18	Yes	Genetic	Absent	Kidney angiolipomas	Normal	No	9M decrease
19	Yes	Genetic	Absent	Skin hypomelanotic macules; Eyes retinal hamartomas	Normal	No	24M no change
20	Yes	Genetic	Present	Skin hypomelanotic macules; Eyes retinal hamartomas	Mild dysfunction	No	6M decrease
21	No	Genetic	Absent	Absent	Normal	No	36M no change
22	Yes	Genetic	Present	Skin hypomelanotic macules	Mild dysfunction	No	6M decrease

## Discussion

CR is the most common type of fetal primary cardiac tumor, with an incidence of 60%, followed by fibromas, teratomas, myxomas, and hemangiomas (3). Each tumor type displayed distinct features. Teratomas typically originate in the pericardium, with mixed echoes, cystic structures, and possible calcifications, often associated with pericardial effusion. Fibromas and hemangiomas are usually solitary, large, and may or may not have calcifications. Myxomas are typically attached to the atria and move with the cardiac cycle. Malignant neoplasms of the fetal heart are rare (15). In the study, to distinguish CR from other cardiac tumors and reduce selection bias, experienced sonographers conducted rigorous fetal echocardiography. In addition, CR diagnoses were supported by family history, clinical symptoms, and genetic tests, and, in some cases, confirmed through multiple prenatal follow-ups, autopsies, or postpartum assessments by pediatricians. For live births, clinical diagnosis of CR is based on confirmation of TSC or tumor shrinkage or disappearance during follow-up, or prenatal and postnatal MRI and ultrasound diagnosis of CR.

It has been reported that the earliest antenatal sonographic detection of cardiac tumors was observed at 15 weeks of gestation, whereas most cases are described after 24 weeks of gestation (10). In this study, the earliest detection was at 23 weeks of gestation, with a median diagnosis at 29 weeks. The delay is often due to mid-term ultrasound screening at around 22 weeks and cases referred from other hospitals after second-trimester ultrasound screenings, resulting in delayed diagnoses. In general, these tumors have a minimal effect on cardiac function, only a few fetuses (15%) exhibited cardiac complications (7). In previous studies, tumor size strongly correlated with fetal mortality (24). In the present study, fetal cardiac complications were observed in 22.2% of cases, always associated with multiple lesions and larger tumor sizes, but there were no cases of fetal death in this study. Multiple lesions were prevalent in 83.3% of fetuses, primarily in the left ventricular wall, right ventricular wall, and interventricular septum, and the number and location of tumors in live infants were consistent with prenatal ultrasound findings, because imaging screening after birth could not detect smaller lesions. Large tumors near the ventricular septum often caused outflow tract obstructions. The results indicated that the number, size and location of the tumor were the main factors leading to intracardiac complications. Moreover, fetal cardiac tumors were sometimes associated with arrhythmias, and the specific type of arrhythmia depended on the tumor's location (25, 26). However, arrhythmias were rare in this study with one case of atrial premature beat without transmitting (presented as bigeminy coupled rhythm, the atrial rate was about 140 beats/min and the ventricular rate was about 70 beats/min) and one case of occasional atrial premature beat, possibly due to the absence of fetal magnetocardiography (FMCG) for accurate electrophysiological assessment of fetal rhythm. The fetus with atrial premature beat without transmitting had a normal postnatal heart rhythm, while the baby with occasional atrial premature beat was terminated.

However, we are more concerned with the relationship between fetal CR and TSC. TSC is a rare autosomal dominant disorder with an incidence of 1/6,800∼1/10,000 live births. It's caused by mutations in the TSC1 or TSC2 genes (27). Detecting intracranial lesions is crucial for diagnosing TSC in fetuses with CRs (28). Although neurological changes are currently difficult to detect in many fetuses with TSC, neuroimaging findings was a much more specific indication of genetic TSC and was proportional to poor prognosis (15). Ultrasound is commonly used for initial imaging, but MRI offers better sensitivity because of MRI provides better contrast between gray and white tissues (29). Previous studies indicated that intracranial nodules of TSC fetuses were rarely detected by ultrasound (11). In our study, intracranial nodules were found in 11 of 35 TSC (31.4%) fetuses by ultrasound with a relatively high detection rate, and combining ultrasound with MRI was the most effective for TSC detection, enhancing the identification of intracranial nodules. Our study highlighted the importance of using both abdominal and transvaginal ultrasound when a fetal heart tumor is identified for accurate diagnosis and prognosis evaluation, this approach improved intracranial nodule detection. And a combination of brain MRI and genetic testing aids in early TSC diagnosis, impacting perinatal management and prognosis.

It is worth mentioning that our study found difference between ultrasound and MRI in detecting fetal intracranial lesions. Previous studies have suggested that subependymal nodules are more easily identified on fetal imaging, however, subcortical lesions are more commonly detected in postnatal brain MRI (10). Our analysis of imaging data found that prenatal ultrasound mostly identifies nodules in the brain parenchyma (90.9%), while prenatal brain MRI primarily detects subependymal nodules (93.3%). For example, ultrasound detected brain parenchyma nodules in case NO. 51, while brain MRI found subependymal nodules (Figure 2). In case NO. 13, prenatal ultrasound identified multiple intracranial nodules, whereas prenatal brain MRI didn't show any abnormalities. However, postpartum ultrasound and neonatal brain MRI revealed intracranial nodules (Figure 4). These phenomena suggested that ultrasound might detect specific fetal brain lesions not visible on MRI, warranting further investigation.

Although both single and multiple CRs were listed as the primary diagnostic criteria for TSC (23), the probability of a diagnosis of TSC varied according to the number of CRs. The presence of multiple CRs is considered a strong predictor of prenatal TSC, with a 95% risk of TSC diagnosis (19, 30). In our study, the TSC group had more multiple intracardiac lesions then simple CR group (*p* = 0.007), aligning with prior research. Notably, the maximum tumor diameter didn't differ significantly between the TSC group and simple CR group (*p* = 0.672). Therefore, multiple CRs has predictive value in the diagnosis of TSC, while the maximum tumor diameter has no significance. In some cases, autopsy revealed more tumors than prenatal ultrasound, which small lesions (1–2 mm) in the ventricular wall went undetected during ultrasound. This underscores the importance of comprehensive scanning to ensure accurate lesion count, particularly in cases of CR with TSC, vital for TSC diagnosis and prognosis assessment.

While TSC is typically an autosomal dominant disorder, most cases result from *de novo* mutations (8, 13). Only 4 fetuses (11.4%) diagnosed with TSC in this study had a family history, family history plays a minor role in TSC diagnosis. Therefore, prenatal diagnosis and prognosis assessment are critical for pregnant individuals and their families. While imaging provides a valuable reference for prenatal TSC diagnosis, genetic sequencing of TSC1/2 exons has been used for a more definitive diagnosis. However, due to the cost and invasiveness of genetic testing, some pregnant individuals opt for termination without genetic testing, so imaging examination is particularly important. In this study, we sought to investigate the efficacy of ultrasound combined with MRI in diagnosing TSC, the result shown that 23 of the 30 fetuses undergoing both prenatal brain MRI and ultrasound were diagnosed with TSC, and 69.6% (16/23) of TSC cases were diagnosed using a combination of ultrasound and MRI, highlighting the sensitivity of this approach. Nevertheless, imaging cannot screen all fetuses with TSC, genetic sequencing remains a recommended method for detecting fetal CR and improving early TSC diagnosis, aiding in perinatal management and prognostic guidance.

Rhabdomyoma cells are glycogen-rich atypical cardiomyocytes, and most of their cytoplasm undergoes vacuolar degeneration and apoptosis after birth so that the tumor will slowly disappear (19). This trend may be influenced by changes in maternal hormone levels and may be related to the pathological basis of CRs. Among 22 live births in the study, CRs of 81.8% (18/22) cases disappeared or shrank after birth, and there was no significant difference in tumor changes between the TSC group and the simple CR group. There were 4 cases with no change in tumor volume, but this may not be the final result because most of the children did not adhere to long-term regular cardiac ultrasonography follow up after birth, the longest follow-up time of echocardiography was only 36 months (3–36 months). In addition, during regular follow-up during pregnancy, we observed that CRs were generally small, slow-growing, and almost unchanged during the third trimester, which was consistent with the self-limited growth pattern of CR. These features are helpful for ultrasound diagnosis of CR.

TSC often involves the central nervous system (CNS), causing epilepsy, cognitive issues, and developmental delays (31–33). Epilepsy is the most prominent neurologic feature in patients with TSC, occurring in 70%–90% of patients and usually occurring within the first year of life (23, 33). In this study, 10 of the 12 surviving TSC-diagnosed children had epilepsy and all occurred within the first year of life, and 4 had developmental delays, and 3 had growth retardation. TSC can also lead to skin issues, retinopathy, and problems with organs such as the kidney and liver (23). In this study, skin hypomelanotic macules were found in 9 cases, retinal hamartoma in 3 cases, retinal pigment degeneration in 1 case, multiple kidney renal cysts in 2 cases, and kidney angiolipomas in 1 case. Skin hypomelanotic macules were very common in children with TSC. The poor prognosis of children with TSC puts tremendous pressure on their families. For children without TSC, a positive prognosis was observed during 6 months to 6 years of follow-up, with no TSC-related symptoms. Patients with simple CR without TSC had better outcomes than those with TSC. Because of the difference in prognosis between them, prognostic counselling for pregnant women with CR should include not only the prognosis of the tumor itself but also the relationship between CR and TSC and the prognosis of TSC, emphasizing the importance of thorough prenatal imaging evaluation and genetic analysis.

### Limitation

Several study limitations warrant consideration. Firstly, there were instances of insufficient pathological data for certain cardiac tumors, and the absence of autopsy information. Secondly, the relatively short follow-up period for echocardiography in some live births may not have fully depicted postnatal changes in cardiac tumors. Lastly, the study did not analyze the prevalence of atrial septal defects (ASD) as comprehensive neuropsychological (NP) tests were not conducted for live births.

## Conclusions

The characteristics of CRs in fetuses play a significant role in assessing fetal cardiac complications, particularly with TSC. Multiple CRs are a significant risk factor for TSC, irrespective of their size or location. Detecting small CRs via ultrasound can be challenging, potentially affecting prognosis assessment. Fetal heart enlargement and enhanced myocardial scanning are helpful for prognosis assessment. Ultrasound and MRI can reveal CRs with intracranial nodules and suggest TSC, though their diagnostic accuracy is limited. TSC gene sequencing is essential for a definitive diagnosis. CRs typically regress or shrink after birth, resulting in a good prognosis for children with isolated CRs. However, children with concurrent TSC often face a less favorable prognosis due to conditions like epilepsy and neurological abnormalities.

## Data Availability

The original contributions presented in the study are included in the article/Supplementary Material, further inquiries can be directed to the corresponding authors.
